# Luteolin Isolated from the Medicinal Plant *Elsholtzia rugulosa* (Labiatae) Prevents Copper-Mediated Toxicity in β-Amyloid Precursor Protein Swedish Mutation Overexpressing SH-SY5Y Cells

**DOI:** 10.3390/molecules16032084

**Published:** 2011-03-02

**Authors:** Rui Liu, Fanrui Meng, Li Zhang, Ailin Liu, Hailin Qin, Xi Lan, Lin Li, Guanhua Du

**Affiliations:** 1 Institute of Materia Medica, Chinese Academy of Medical Sciences and Peking Union Medical College, Beijing 100050, China ; E-Mail: liurui@imm.ac.cn (R.L.); 2 Xuanwu Hospital Capital Medical University, Beijing 100053, China

**Keywords:** *Elsholtzia rugulosa*, luteolin, Alzheimer’s disease, amyloid-β peptide, copper

## Abstract

Luteolin, a 3’,4’,5,7-tetrahydroxyflavone, is a plant flavonoid and pharmacologically active agent that has been isolated from several plant species. In the present study, the effects of luteolin obtained from the medicinal plant *Elsholtzia rugulosa* and the related mechanisms were examined in an Alzheimer's disease (AD) cell model. In this model, copper was used to exacerbate the neurotoxicity in β-amyloid precursor protein Swedish mutation stably overexpressed SH-SY5Y cells (named “APPsw cells” for short). Based on this model, we demonstrated that luteolin increased cell viability, reduced intracellular ROS generation, enhanced the activity of SOD and reversed mitochondrial membrane potential dissipation. Inhibition of caspase-related apoptosis was consistently involved in the neuroprotection afforded by luteolin. Furthermore, it down-regulated the expression of AβPP and lowered the secretion of Aβ_1-42_. These results indicated that luteolin from the *Elsholtzia rugulosa* exerted neroprotective effects through mechanisms that decrease AβPP expression, lower Aβ secretion, regulate the redox imbalance, preserve mitochondrial function, and depress the caspase family-related apoptosis.

## 1. Introduction

Alzheimer's disease (AD) is a neurodegenerative disorder characterized by the progressive loss of memory and cognitive decline. Intracellular neurofibrillary tangles and extracellular senile plaques, mainly composed of different amyloid-β (Aβ) species, are the major pathological hallmarks of AD [1]. Aβ is generated from the amyloid precursor protein (APP) by enzymatic cleavage involving β-secretase and the γ-secretase complex [2,3,4], but the causes leading to the development of Aβ are not well understood. It has been suggested that metal ions such as copper, iron, zinc, and the exogenous contaminant aluminum are involved in the assembly and neurotoxicity of Aβ species [5,6,7,8,9]. In particular, copper, iron and zinc dyshomeostasis were evident within AD-affected brain [10,11]. In the related metal ion dyshomeostasis, high concentrations of copper have been found within the amyloid deposits. APP and Aβ both have a copper binding domain (CuBD) [12,13,14]. Cu(II) serves as cofactor to Aβ peptides and APP protein, facilitating the formation of Aβ aggregates, influencing their conformational transformation, and exacerbating the oxidative stress processes [12,15], which yield highly toxic reactive oxygen species and trigger the cascade of biochemical alterations eventually leading to neuronal cell death [16,17].

*Elsholtzia rugulosa* (Labiatae), which is distributed in the Sichuan, Yunnan and Guizhou provinces of China, is known as a herbal tea, medicinal herb and honey plant [18]. In these regions, the title plant is widely used by local people in the treatment of colds, headaches, pharyngitis, coughs and fever [19]. Flavonoids such as luteolin are ubiquitous plant secondary metabolites and have a variety of biological effects, including antioxidant, anti-inflammatory, anti-AP1 activation and phytoestrogen-like activities [20,21,22]. Some evidence has shown that luteolin had anti-amnesic effects both *in vitro* and *in vivo* [23,24]. Our previous studies have demonstrated that luteolin exerted anti-amnesic effects in mice [25], and that it played an essential role against Aβ_25-35_-induced toxicity in cerebral microvascular endothelial cells [26]. Even though there is evidence suggesting that luteolin exerted protective effects in AD models [23,24,25,26,27], no preexisting study has been reported the neuroprotective activity of luteolin isolated from *Elsholtzia rugulosa* (Labiatae). Thus, as a part of our ongoing screening program to evaluate the neuroprotective potential of natural compounds, we investigated the *in vitro* neuroprotective activity of *Elsholtzia rugulosa* through activity-guided fractionation. Subsequently, the effects of luteolin isolated from *Elsholtzia rugulosa* were evaluated on copper-induced neurotoxicity in the β-amyloid precursor protein Swedish mutation stably overexpressing SH-SY5Y cells.

## 2. Results and Discussion

In this study, our findings indicate that copper triggers the neurotoxicity in APPsw overexpressing cells, which exacerbates the Aβ neurotoxicity and can be taken as a model of AD. Luteolin treatment exerted neuroprotection through mechanisms that decrease AβPP expression, lower Aβ secretion, regulate the redox imbalance, preserve mitochondrial function, and depress caspase family-related apoptosis.

Transfection of an APP gene into a cell to generate Aβ has been used as a classical AD model. Metal ion dyshomeostasis is a well-recognized cofactor in AD [28]. In order to better mimic the *in vivo* Aβ-induced neurotoxicity, instead of directly using single Aβ treatment, we used a copper-treated APPsw overexpressing cell system, which could link well metal ion imbalance and Aβ aggregation and toxicity. Elevation of oxidative stress, dysfunction of mitochondrial membrane and caspase-related apoptosis were seen in copper-treated APPsw cells, indicating that copper plays a role on neurotoxicity in this model.

### 2.1. Luteolin Increased Cell Viability against Copper-Induced Toxicity in APPsw Overexpressing Cells

**Figure 1:**
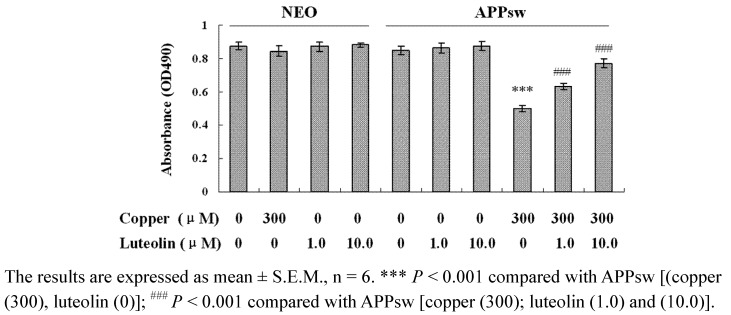
Effects of luteolin on neo and APPsw cell viability. The neo and APPsw cells were treated by different methods and cell viability was measured by the MTS assay.

As shown in Figure 1, no significant difference in cell viability is seen among the neo cells and APPsw cells, and cell viability does not show any significance among the neo cells treated with luteolin. However, cell viability in APPsw cells is significantly decreased in the presence of 300 μM copper (*P* < 0.001), while no effect is seen in neo cells with the same treatment. Luteolin enhances the cell viability at 1.0 μM and 10.0 μM after being exposed to 300 μM copper (*P* < 0.001). Luteolin did not show significant effect in the APPsw cells without copper treatment.

### 2.2. Luteolin Treatment Decreased AβPP Expression and Aβ Secretion in APPsw Overexpressing SH-SY5Y Cells

**Figure 2:**
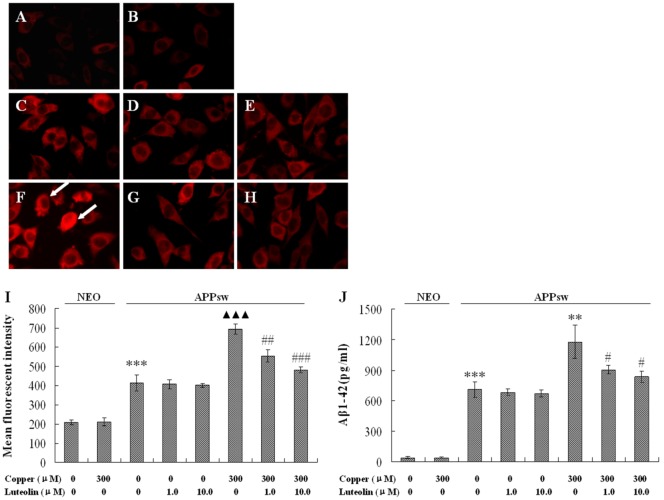
Effects of luteolin on AβPP expression and Aβ_1-42_ in culture medium of the neo and APPsw cells. Data are presented as mean ± S.E.M., n = 4. Images present treatment with (**A**) neo group; (**B**) neo group being exposed to 300 μM copper for 24 h; (**C**) APPsw group; (**D**) APPsw group with luteolin treatment at 1.0 μM; (**E**) 10 μM for 24 h; (**F**) APPsw group being exposed to 300 μM copper for 24 h; (**G**) APPsw group being exposed to 300 μM copper with luteolin treatment at 1.0 μM; (**H**) 10.0 μM for 24 h. (**I**) Fluorescent intensity of AβPP staining, *** *P* < 0.001 compared with neo cells [(copper (0), luteolin (0)], ^▲▲▲^ *P* < 0.001 compared with APPsw [(copper (0), luteolin (0)], ^##^ *P* < 0.01 and ^###^ *P* < 0.001 compared with APPsw [copper (300); luteolin (0)]. (**J**) Aβ_1-42_ contents in culture media, *** *P* < 0.001 compared with neo cells [(copper (0), luteolin (0)], ** *P* < 0.01 compared with APPsw [(copper (0), luteolin (0)], ^#^ *P* < 0.05 compared with APPsw [copper (300); luteolin (0)].

Evidence has shown that copper is able to interact with both Aβ and APP, and that AβCu^2+^ and APPCu^2+^ are both toxic to the cells [29]. In the present study, the AβPP level and the Aβ generation were both elevated by 300 μM copper treatment. Copper increased the expression of AβPP almost by one fold (*P* < 0.001, Figure 2A), and Aβ_1-42_ were measured over 16.8 fold higher in the APPsw cell medium, and much higher after being exposed to copper – over 27.99 fold that of the neo cells (*P* < 0.01, Figure 2B). Luteolin blocked the increased expression of AβPP exacerbated by copper at 1.0 μM and 10.0 μM (*P* < 0.01–0.001, Figure 2A). Aβ_1-42_ peptide secretion was also significantly decreased by 23.3% and 28.9% at 1.0 μM and 10.0 μM, respectively (*P* < 0.05, Figure 2B), but luteolin does not show these effects in APPsw cells without copper treatment.

Luteolin is a flavonoid, and possesses the property of binding to transition metal ions [30,31]. Analysis of the activity-structure relationships of luteolin and related compounds indicate that the presence of the C2-C3 double bond on the C ring and possession of both the catechol group in the B-ring and the 3-hydroxyl group are critical for this biological activity. In particular, the copper reducing activity seems to depend largely on the number of hydroxyl groups. Additionally, other evidence suggests that luteolin reduces soluble Aβ_1-40, 42_ isoforms [32] and inhibits Aβ fibril formation [33], but in the present study, the Aβ level was not lowered in culture medium of APPsw cells by single luteolin treatment. Thus, it is deduced that neuroprotection of luteolin may be partly explained to the binding to copper ions to reduce the up-regulation of the APP level and decrease the secretion of Aβ peptides, instead of the influence of Aβ fibril formation.

### 2.3. Luteolin Treatment Regulated the Redox Imbalance in APPsw Overexpressing SH-SY5Y Cells

**Figure 3:**
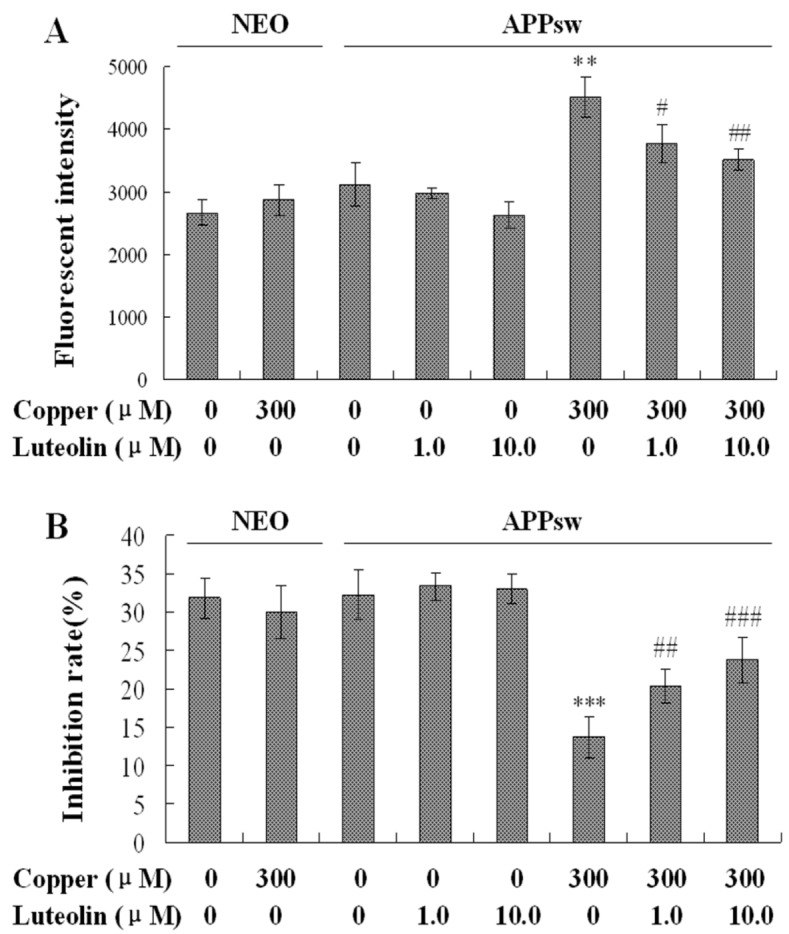
Effects of luteolin on intracellular ROS accumulation and SOD activity of the neo and APPsw cells. The result is expressed in mean ± S.E.M., n = 5. (**A**) ROS generation in the neo and APPsw cells. ** *P* < 0.01 compared with APPsw [(copper (0), luteolin (0)]; ^#^ *P* < 0.05 and ^##^ *P* < 0.01 compared with APPsw [copper (300); luteolin (0)]. (**B**) SOD activity in the neo and APPsw cells. *** *P* < 0.001 compared with APPsw [(copper (0), luteolin (0)]; ^##^ *P* < 0.01 and ^###^ *P* < 0.001 compared with APPsw [copper (300); luteolin (0)].

Copper is one of the redox metals, leading to increased oxidative stress (with the production of excess superoxide and hydroxyl radicals) [34], and is associated with the severe redox imbalance in this cell model. In the present study, copper increased ROS generation by about 1.5 fold (*P* < 0.01, Figure 3A), and decreased the SOD activity to about 40% in the APPsw cells (*P* < 0.001, Figure 3B).

Luteolin has been demonstrated to possess free radical scavenging activity against hydrogen peroxide and ROS [35]. In good agreement with this notion, we observed that in the present model luteolin scavenged ROS generation to protect the cells at the concentrations of 1.0 and 10 μM (*P* < 0.05–0.01, Figure 3A). Besides the fact that luteolin scavenged the excrescent ROS, it also increased the antioxidative capacity in the cell model. The inhibition rate of SOD indicated the scavenging ability of the cell, and after cultured with luteolin, the activity of SOD increased (*P* < 0.01–0.001, Figure 3B). Thus, the present data showed that luteolin provides sufficient antioxidant effect through scavenging the ROS and ameliorating the antioxidative ability in APPsw cells.

### 2.4. Luteolin Treatment Depressed the Apoptosis Stress in APPsw Overexpressing SH-SY5Y Cells

As a kind of neurodegenerative disease, AD is marked by a special neuron apoptosis. Several mechanisms are responsible for the elevation of apoptosis, one of which is mitochondrial dysfunction [36,37]. Loss of mitochondrial membrane potential, as a symbol of mitochondrial dysfunction, contributes to cell death by increasing the production of ROS, and releasing the death regulatory and signal molecules from the intermembrane space, thereby leading to caspase-dependent cytotoxicity and downstream apoptotic signaling [38].

**Figure 4:**
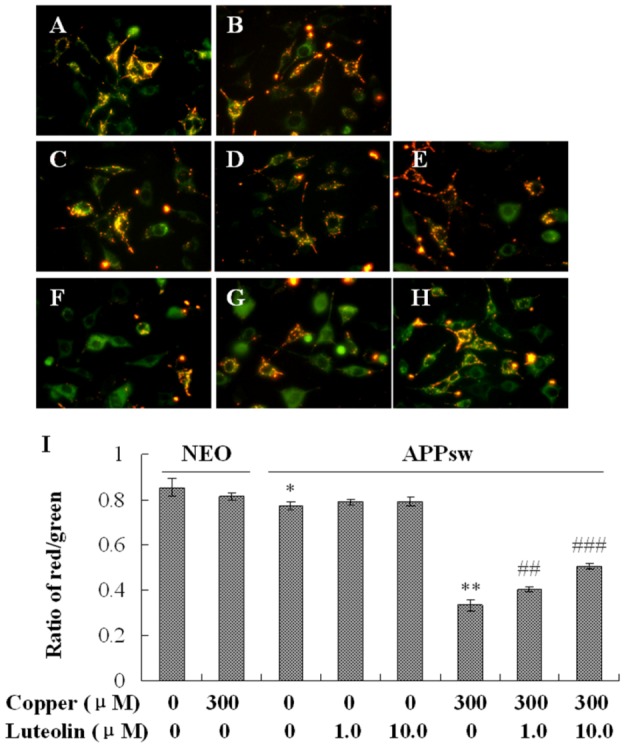
Effects of luteolin on mitochondrial membrane potential of the neo and APPsw cells. Images present treatment with (**A**) neo group; (**B**) neo group being exposed to 300 μM copper for 24 h; (**C**) APPsw group; (**D**) APPsw group with luteolin treatment at 1.0 μM; (**E**) 10 μM for 24 h; (**F**) APPsw group being exposed to 300 μM copper for 24 h; (**G**) APPsw group being exposed to 300 μM copper with luteolin treatment at 1.0 μM; (**H**) 10.0 μM for 24 h. (**I**) Ratio of fluorescent intensity of red/green. Data are presented as mean ± S.E.M., n = 4, * *P* < 0.05 compared with neo cells [(copper (0), luteolin (0)], ** *P* < 0.01 compared with APPsw [(copper (0), luteolin (0)], ^##^ *P* < 0.01 and ^###^ *P* < 0.001 compared with APPsw [copper (300); luteolin (0)].

**Figure 5:**
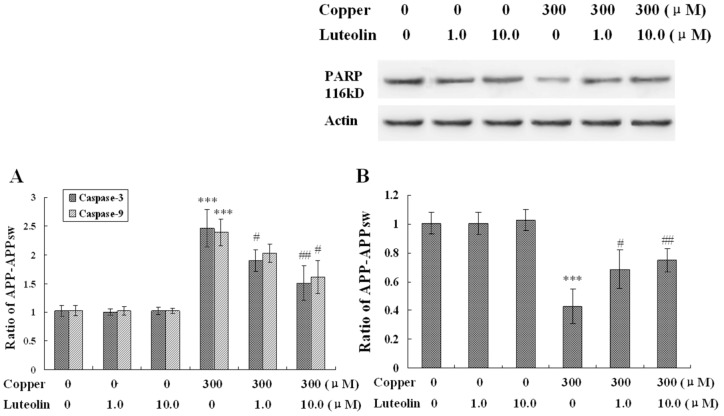
Effects of luteolin on apoptosis stress of the neo and APPsw cells. The result is expressed in mean ± S.E.M., n = 4. (**A**) Activity of caspase-3 and -9 in the neo and APPsw cells. *** *P* < 0.001 compared with APPsw [(copper (0), luteolin (0)]; ^#^ *P* < 0.05 and ^##^ *P* < 0.01 compared with APPsw [copper (300); luteolin (0)]. (**B**) Expression of PARP in the neo and APPsw cells. *** *P* < 0.001 compared with APPsw [(copper (0), luteolin (0)]; ^#^ *P* < 0.05 and ^##^ *P* < 0.01 compared with APPsw [copper (300); luteolin (0)].

In this study, it was found that copper yielded highly toxic ROS, and triggered a cascade of biochemical alterations, marked by mitochondrial membrane potential loss and the sequential activation of caspase-9 and -3. The lipophilic cationic probe JC-1 was used to evaluate mitochondrial membrane potential. There was no difference in fluorescent ratio of red/green among the neo cells in the absence and presence of copper. When the APPsw cells were exposed to copper, the ΔΨm rapidly depolarized, as indicated by the increase in green fluorescence, the concomitant disappearance of red fluorescence and the decreased red/green fluorescent ratio (*P* < 0.01, Figure 4). Treatment with luteolin at 1.0 μM and 10.0 μM for the APPsw cells reduced the changes in ΔΨm as indicated by repression of green fluorescence, restoration of red fluorescence and increase of fluorescent ratio of red/green (*P* < 0.01–0.001, Figure-4). Furthermore, the activities of caspase-3 and caspase-9 were both increased in APPsw cells after the copper treatment (*P* < 0.001, Figure 5A), and these dysfunctions were alleviated remarkably by treatment with luteolin (*P* < 0.05–0.01, Figure 5A).

Another characteristic associated with the execution phase of the apoptosis pathway is the specific poly-(ADP-ribose) polymerase (PARP) cleavage, leading to inactivation of the enzyme, thus preventing futile DNA repair cycles [39]. It has been reported that caspase-3 is the most efficient processing enzyme for PARP [40]. Integration of PARP was examined. As expected, the amount of 116 kDa protein decreased followed upon the over activation of caspase-9 and -3 (*P* < 0.001, Figure 5B). This phenomenon was ameliorated by luteolin treatment (*P* < 0.05–0.01, Figure 5B). Based on the result, it is a reasonable deduction that luteolin preserved mitochondrial function induced by copper, and then, attenuated the damage to the mitochondrial-dependent apoptosis pathway.

## 3. Experimental

### 3.1. Reagents

DCFH-DA, G418, and caspase-3 and capase-9 assay kits were purchased from Sigma–Aldrich Chemical Co. (St. Louis, MO, USA). Cell Titer96 Aqueous MTS assay kit was purchased from the Promega Company (Madison, WI, USA). SOD inhibition assay kit was purchased from Dojindo Laboratory (Kumamoto, Japan). Dulbecco’s modified Eagle’s medium/Ham 12 (DMEM/F12) and fetal calf serum were purchased from Gibco BRL (Grand Island, NY, USA). Rabbit polyclonal antibody for AβPP, PARP and actin were purchased from Santa Cruz Biotechnology (Santa Cruz, CA, USA). All other chemicals were analytical grade and obtained locally in China.

### 3.2. Plant Materials

The whole plant of *Elsholtzia rugulosa* Hemsl. (Labiatae) was collected in the Yunnan Province of China, in September 2005, and identified by Dr. Hailin Qin, Chinese Academy of Medical Sciences. A voucher specimen (ID-14288) was deposited in the Herbarium of the Department of Botany, Institute of Materia Medica, Chinese Academy of Medical Sciences, China.

### 3.3. Extraction and Isolation of Luteolin

The whole plant of *Elsholtzia rugulosa* (27 kg) was air-dried, powdered and then refluxed with 95% EtOH (3 × 4,000 mL, 2 h, 1.5 h and 1.5 h, respectively). The combined EtOH solution was filtered and evaporated under reduced pressure to yield a crude extract (1.675 g) which was dissolved in 80% EtOH (4,000 mL), and extracted with petroleum ether (60~90 °C, 3 × 4,000 mL, 2 h each). The petroleum ether (PE) layer was evaporated. Then, evaporation of the aqueous layer under reduced pressure yielded a brown residue which was dissolved in water (8000 mL), and then extracted with EtOAc (5 × 8, 000 mL, 4 h each) to form an EtOAc extract (140 g). The EtOAc fraction of the plant was subjected to silica gel chromatography with CHCl_3_-MeOH (25:1, R_f_ = 0.67) as the eluent to yield the *Elsholtzia rugulosa* luteolin. The structure of the compound was determined by its physico-chemical and spectral data (LC-MS, 1D and 2D NMR), which were in agreement with those reported in the literature [41,42]. One hundred and thirty three (133) mg of luteolin were obtained and the purity of the compound was 98%.

### 3.4. Cell Cultures, Transfection and Treatments

Human neuroblastoma SH-SY5Y cells were grown in DMEM/F12 supplemented with 10% fetal calf serum at 37°C in humidified 5% CO_2_ air. Stably transfected SH-SY5Y cell lines expressing human APPsw or empty vector (neo) pCLNCXv.2 were made by using FuGENE HD transfection reagent (Roche Diagnostics GmbH. Roche Applied Science, Mannheim, Germany) and selected by G418 resistance. Cells were randomly divided into eight groups: (1) neo group; (2) neo group being exposed to 300 μM copper for 24 h; (3) APPsw group; (4) APPsw group with luteolin treatment at 1.0 μM; (5) 10 μM for 24 h; (6) APPsw group being exposed to 300 μM copper for 24 h; (7) APPsw group being exposed to 300 μM copper with luteolin treatment at 1.0 μM; (8) 10.0 μM for 24 h.

### 3.5. MTS Assay for Cell Viability

Cell survival rates were assessed by the MTS [3-(4,5-dimethylthiazol-2-yl)-5-(3-carboxymethoxy- phenyl)- 2-(4-sulfophenyl)-2H-tetrazolium, inner salt] assay (Promega, Madison, WI, USA) according to the manufacturer’s protocol and detected using a SpectraMax Plus microplate reader (Molecular Devices Corp., Sunnyvale, CA, USA).

### 3.6. Mitochondrial Membrane Potential (ΔΨm) Detection

Loss of ΔΨm was measured by staining with the fluorescent probe JC-1. Cells in each group were incubated in the medium containing 5 μg/mL JC-1 after being exposed to copper and treated with luteolin. Fluorescent images were acquired with a digital camera mounted on an Olympus IX71 fluorescent microscope. When excited at 488 nm, the fluorescence emission of JC-1 was measured at wavelengths corresponding to its monomer (530 ± 15 nm) and J aggregate (> 590 nm) forms. Fluorescent intensity was measured in a SpectraMax Plus microplate reader.

### 3.7. Measurements of Intracellular ROS and SOD

Production of reactive oxygen species (ROS) was monitored spectrofluorometrically by the 2', 7'-dihydrodichlorofluorescein diacetate (DCFH-DA) assay with some modifications [43]. DCFH-DA was added to the culture plates at a final concentration of 5 μM and incubated for 40 min at 37 °C in darkness. DCF fluorescence intensity was detected with emission wavelength at 535 nm and excitation wavelength at 485 nm using a SpectraMax Plus microplate reader.

After being exposed to copper and/or different concentrations of luteolin, cells were collected by scraping and low-speed centrifugation (1,000 rpm, 10 min). The supernatants were crushed by sonication (60W with 0.5 s interval for 15 min), and then centrifuged at 10,434 rpm for 15 min. The supernatants were used to measure the cellular SOD with WST-1 based SOD inhibition assay (Kumamoto, Japan). The solutions in each well were added as described in the manufacturer’s protocol, and the microplate was stirred thoroughly and then incubated at 37 °C for 20 min. The absorbance at 440 nm of the endpoint reaction was measured by using a SpectraMax Plus microplate reader. Percentage inhibition of each sample was calculated using following equation: {[(*A*_1_ −*A*_3_)−(*A*_S_ −*A*_2_)]/ (*A*_1_ −*A*_3_)}×100, where *A*_1_, *A*_2_, *A*_3_ and *A*_S_ were the absorbance at 440 nm for uninhibited test, blank sample, blank reagent and sample, respectively.

### 3.8. β-APP Immunostaining Analysis

β-APP was examined by indirect immunofluorescence assay. Briefly, after copper and luteolin treatments, cells were fixed with 4% paraformaldehyde for 30 min at 4 °C, permeabilized with 0.3% Triton X-100 for 10 min, and blocked with 3% BSA for 30 min at room temperature. Next, cells were incubated with primary anti-β-APP antibody overnight at 4 °C, followed by Cy3-conjugated secondary antibody.

### 3.9. ELISA Assay for Aβ_1-42_ in Medium

Aβ_1-42_ contents were measured in cultures and medium using ELISA. After copper and luteolin treatments, the culture medium was collected and centrifuged (1,000 rpm) at 4 °C for 5 min. The supernatants were used for the determination of quantitative levels of Aβ_1-42_ according to the ELISA Kit manufacturer’s instruction (Jingmei Biotech, China). The optical density was measured using the SpectraMax Plus microplate reader at 450 nm, and values obtained from standard curves generated with the limits of detection of 5 pg/mL for Aβ_1-42_.

### 3.10. Measurement of Activity of Caspase-3 and Caspase-9

Measurement of activity of caspase-3 and capase-9 in the cells was performed using the commercially available caspase-3 and caspase-9 assay kits. The colorimetric assays were based on the hydrolysis of the Ac-DEVD *p*-nitroaniline by caspase-3 and Ac-LEHD *p*-nitroaniline by caspase-9, resulting in the release of the *p*-nitroaniline moiety. Proteolytic reactions were carried out in extraction buffer containing 200 μg of cytosolic protein extract and 40 μM Ac-DEVD *p*-nitroaniline or 40 μM Ac-LEHD *p*-nitroaniline. The reaction mixtures were incubated at room temperature for 2 h, and the formation of *p*-nitroaniline was measured at 405 nm on the SpectraMax Plus microplate reader. The concentration of the *p*-nitroaniline released from the substrate was calculated from the absorbance values.

### 3.11. Western Blot Analysis

For Western blotting, cells were washed in ice-cold PBS and scraped in the lysis buffer [Tris-HCl (20 mM, pH 7.6), 150 mM NaCl, 1 mM of EGTA, EDTA and phenylmethylsulfonyl fluoride (PMSF), 1% NP-40, 0.1% aprotinin, 0.7 mg/mL pepstatin, and 1 μg/mL leupeptin.]. The lysate was sonicated and then centrifuged at 11,430 rpm for 10 min at 4 °C. Cell lysates were denatured at 95 °C in sample buffer (60 mM Tris, pH 6.8, 2% SDS, 25% glycerol, 0.1% bromophenol blue, 20% β-mercaptoethanol) and processed for SDS-polyacrylamide gel electrophoresis (SDS-PAGE) and electrotransferred to a PVDF membrane (Millipore, Bedford, MA). Immunoblottting was done in 5% nonfat dry milk in Tris-buffered saline. Antibodies used were anti-PARP and anti-actin. Immunoreactivity was detected using peroxidase-conjugated goat anti-rabbit IgG. The protein bands were visualized using an ECL^TM^ detection kit (GE Healthcare) and exposure to X-ray films. Relative optical densities and areas of bands were quantified using an image densitometer.

### 3.12. Statistical Analysis

Data are expressed as mean ± S.E.M. Each experiment was repeated at least three times. One-way ANOVA followed by Student’s two-tailed unpaired *t*-test was used for the statistical analysis by employing SPSS 11.0 software. Differences were considered significant at *P* < 0.05.

## 4. Conclusions

This study deals with the effects of luteolin from *Elsholtzia rugulosa* and its mechanisms of action in an AD cell model. Based on this model, we demonstrated that luteolin increased cell viability, reduced intracellular ROS generation, enhanced the activity of SOD and reversed mitochondrial membrane potential dissipation. Luteolin also inhibited caspase-related apoptosis, and furthermore, down-regulated the expression of AβPP and lowered the secretion of Aβ_1-42_. These results indicated that luteolin from *Elsholtzia rugulosa* could exert neroprotection through the mechanisms that decrease AβPP expression, lower Aβ secretion, regulate the redox imbalance, preserve mitochondrial function, and depress the caspase family-related apoptosis.
